# Regulatory T cells in the peripheral blood of women with gestational diabetes: a systematic review and meta-analysis

**DOI:** 10.3389/fimmu.2023.1226617

**Published:** 2023-12-04

**Authors:** Hania Arain, Tina Patel, Nicoleta Mureanu, Athina Efthymiou, Giovanna Lombardi, Timothy Tree, Kypros H. Nicolaides, Panicos Shangaris

**Affiliations:** ^1^ Department of Women and Children’s Health, School of Life Course and Population Sciences, Faculty of Life Sciences and Medicine King’s College London, London, United Kingdom; ^2^ Harris Birthright Research Centre for Fetal Medicine, King’s College Hospital, London, United Kingdom; ^3^ Peter Gorer Department of Immunobiology, School of Immunology & Microbial Sciences, Faculty of Life Sciences & Medicine, King’s College London, London, United Kingdom

**Keywords:** gestational diabetes mellitus (GDM), regulatory T-cells (Tregs), immune dysregulation, chronic low-grade inflammation, treg markers, systematic review & meta-analysis

## Abstract

**Background:**

Gestational diabetes (GDM) affects approximately 14% of pregnancies globally and is associated with short- and long-term complications for both the mother and child. In addition, GDM has been linked to chronic low-grade inflammation with recent research indicating a potential immune dysregulation in pathophysiology and a disparity in regulatory T cells.

**Objective:**

This systematic review and meta-analysis aimed to determine whether there is an association between GDM and the level of Tregs in the peripheral blood.

**Methods:**

Literature searches were conducted in PubMed, Embase, and Ovid between the 7th and 14th of February 2022. The inclusion criteria were any original studies published in the English language, measuring differentiated Tregs in women with GDM compared with glucose-tolerant pregnant women. Meta-analysis was performed between comparable Treg markers. Statistical tests were used to quantify heterogeneity: *τ* ^2^, *χ* ^2^, and *I* ^2^. Study quality was assessed using a modified version of the Newcastle-Ottawa scale.

**Results:**

The search yielded 223 results: eight studies were included in the review and seven in the meta-analysis (GDM = 228, control = 286). Analysis of Tregs across all trimesters showed significantly lower Treg numbers in women with GDM (SMD, −0.76; 95% CI, −1.37, −0.15; *I* ^2^ = 90%). This was reflected in the analysis by specific Treg markers (SMD −0.55; 95% CI, −1.04, −0.07; *I* ^2^ = 83%; third trimester, five studies). Non-significant differences were found within subgroups (differentiated by CD4^+^FoxP3^+^, CD4^+^CD127^−^, and CD4^+^CD127^−^FoxP3) of both analyses.

**Conclusion:**

GDM is associated with lower Treg numbers in the peripheral maternal blood. In early pregnancy, there is clinical potential to use Treg levels as a predictive tool for the subsequent development of GDM. There is also a potential therapeutic intervention to prevent the development of GDM by increasing Treg populations. However, the precise mechanism by which Tregs mediate GDM remains unclear.

**Systematic review registration:**

https://www.crd.york.ac.uk/prospero, identifier CRD42022309796.

## Introduction

Gestational diabetes mellitus (GDM), characterized by glucose intolerance with first onset during pregnancy, is a common obstetric disease affecting up to 14% of pregnancies globally, with incidence varying between and within countries (1–3). The condition is associated with both short- and long-term complications for the mother and child. Adverse perinatal and neonatal outcomes include increased risk of hypertensive disorders (including pre-eclampsia), stillbirth, preterm birth, cesarean and operative vaginal delivery, macrosomia, shoulder dystocia, neonatal hypoglycemia, and hyperbilirubinemia. Long-term complications include an increased risk of type 2 diabetes in both mother and child as well as metabolic syndrome and cardiovascular disease in women and obesity in children (itself a risk for a range of diseases) (4–6).

The mechanisms involved in the pathophysiology of GDM are complex and not fully understood. They include failure of pancreatic β cells, oxidative stress, and adipose expandability. Recent evidence also suggests an association with a maternal state of chronic low-grade inflammation where there is immune dysregulation through enhanced T-cell activation and increased circulating levels of proinflammatory cytokines (7, 8).

### Underlying pathophysiology–immune implications: Tregs

Regulatory T cells (Tregs) are a specialized subset of CD4^+^ T cells responsible for moderating tolerance to self-antigens and play a part in regulating the T-cell-mediated inflammatory response (9). Tregs are defined by their cell surface markers CD4^+^, CD25^+^, CD127^−^, and FoxP3^−^, whose expression is understood to facilitate Treg development (10). The mechanisms of their immunosuppressive function include cell-to-cell contact, releasing anti-inflammatory cytokines such as IL-10 and TGF-β, directly inducing apoptosis in the antigen-presenting cell (APC) and consuming IL-2 to reduce T-cell proliferation (11). In pregnancy, the immune system is known to play a complex and dynamic role, switching from proinflammatory to anti-inflammatory to proinflammatory (12). Moreover, Tregs have been evidenced to increase during the first two trimesters, before a non-significant decrease in the third trimester (13).

### Current research on Tregs in GDM and added value of this study

Dysregulation of Tregs, mostly lower levels, has been associated with several adverse obstetric conditions, including recurrent miscarriage, pre-eclampsia (14–16), and diabetes mellitus (17, 18). Importantly, the loss of Treg function in immunodysregulation polyendocrinopathy X-linked syndrome (IPEX) leads to insulin-dependent diabetes mellitus (19).

#### Rationale

Despite much interest in Tregs, research focusing on Treg populations and activity in GDM is relatively limited with only a few studies conducted in this area (20–27). Additionally, the synthesis of these findings related to Tregs in pregnancy, in the form of a systematic review and meta-analysis, has not previously been performed.

This systematic review and meta-analysis aims to determine whether there is an association between Treg numbers in the peripheral maternal blood and the development of GDM. The primary objective is therefore comparing the overall proportion of Tregs between patients with GDM and those with healthy pregnancies. This may indicate the future potential prognostic (the possibility of predicting later development of GDM based on Treg populations early in pregnancy) and therapeutic use of Tregs (potential to target said populations) when approaching GDM diagnosis and management in the future.

## Methods

This systematic review and meta-analysis was registered on PROSPERO on the 10th of February 2022 following the completion of the protocol form (CRD42022309796) (available from https://www.crd.york.ac.uk/prospero/display_record.php?ID=CRD42022309796).

Broadly using the PICOS framework, the primary outcome was to determine whether there is an association between the overall proportion of Tregs in the peripheral blood of women with GDM when compared with glucose-tolerant pregnant women of a similar gestational age.

### Inclusion/exclusion criteria

The inclusion criteria were original human studies (all study designs) measuring Treg levels in the peripheral blood of pregnant women with GDM, published in the English language. Unpublished literature, abstracts, and literature reviews were excluded as well as studies of participants with previous immune dysregulation (e.g., studies of women with HIV), samples taken from cord blood or decidua, and studies with ambiguity in the methodology of identifying Tregs.

### Search

The search was electronically conducted by two reviewers, between the 7th and 14th of February 2022, using the following databases: Embase (using the Ovid search software), Medline, and PubMed. To ensure that all potentially relevant studies were included, the search strategy used a combination of keywords, appropriate variants, and all relevant terms. The authors remained vigilant for new publications. The exact search words used are listed in Appendix 1.

Contact was attempted but not established with the authors. Summary estimates were sought for the meta-analysis of the desired groups, while any relevant individual data were extracted for the review. Search results were compiled in a shared Google Drive. Duplicates were identified and highlighted using the “highlight duplicates” in the Google Sheets feature, and the remaining titles and abstracts were screened for relevance independently by each reviewer. A traffic light system was used to sort studies according to the eligibility criteria. Discrepancies were independently resolved by a third reviewer.

### Data analysis

The Cochrane data extraction and assessment template was used as the basis to design review-specific data extraction categories (28). Data were collected for the following subcategories: study demographics (country, year, methodology, inclusion/exclusion criteria, Treg markers and methodology used, populations, follow-up, etc.), maternal data (gestational age at sample collection, age, weight/BMI, glucose levels, gestational age at delivery where applicable), child outcomes, and all data relevant to Tregs (including expression markers and T-cell populations where available). Data extraction for studies that presented data in a graphical format without access to a numerical summary was done using WebPlotDigitizer 4.5 (29). All extracted data were cross-checked to minimize error.

Meta-analysis was performed across studies that used comparable Treg cell markers to identify populations. This was defined as the Treg markers denoted by previous literature to be required in identifying Treg cells: CD25, CD127, and Foxp3 (30).

The measure of effect for quantitative analysis was the standardized mean difference (SMD) of Treg populations. For studies where data were presented as median and range/interquartile range, an online tool (“Mean-Variance Estimation”) was used to convert the data to mean and standard deviation. This tool is specifically designed for statistical conversion when performing meta-analysis using the methods of Luo et al. and Wan et al. (31, 32). Using the statistical formula, any study using mean and standard error of the mean (SEM) was also converted to mean and standard deviation. For studies where patients with GDM were grouped separately, the mean and standard deviation (SD) of the groups were combined using the formula (Appendix 2) in the Cochrane Handbook (33).

Statistical analyses were performed using RevMan software version 5.4.1 with data reported with 95% confidence intervals (CIs). Random-effects models were used to determine the summary effect estimate in studies with high heterogeneity as recommended by the Cochrane Handbook (Higgins et al., 2022). Graphical representation of analysis was also used to produce forest plots. Tau-squared (*τ* ^2^), chi-squared (*χ* ^2^), and inconsistency index (*I* ^2^) tests were used to quantify heterogeneity between studies.

With consideration to Treg markers and Treg profiles dependent on gestational age, data from the studies were analyzed according to two main considerations: analysis by trimester for all studies and analysis by Treg markers for studies using samples in the third trimester to account for potential sources of heterogeneity due to gestational age. In studies that measured multiple markers (differentiated in data), analysis was done according to the most appropriate measure in comparison to other studies. Further sensitivity analysis was also performed excluding two studies with a population of only overweight women where populations were of a BMI greater than *x* as high BMI has been linked to dysregulation to explore reasons for heterogeneity (34).

### Risk of bias

Quality assessment was done using a modified version of the Newcastle-Ottawa scale (NOS) as recommended by Cochrane (33). The full criteria is provided in Appendix 3.

## Results

### Search results and study selection

**Figure 1:**
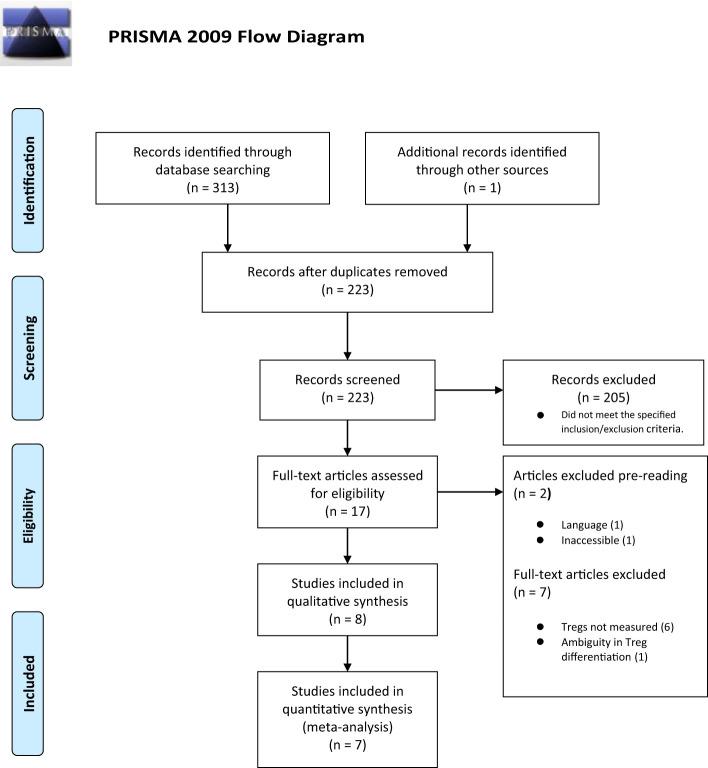
PRISMA flow diagram.

Three hundred and thirteen titles were assessed for eligibility including one study published after the main search was conducted (26). There were 91 duplicates, and 305 titles were excluded. **Figure 1** summarizes the selection process with full reasons for exclusion defined in Appendix 4.

Data from eight studies were included in the systematic review (GDM = 254, control = 309), and data from seven studies were included in the meta-analysis (GDM = 228, control = 286); the study by Sifnaios et al., 2019 was excluded from quantitative analysis because the authors only measured Tregs based on staining of peripheral T cells for the expression of the specific intracellular cytokine IL-10 instead of the predefined Treg markers determined in the methods.

### Study characteristics

All studies were observational with populations recruited from hospital settings: five were case–control studies (21–24, 27) and three were cohort studies (20, 25, 26). Five studies included a defined inclusion criteria, while three studies did not have a defined exclusion criteria. Patients with systemic and autoimmune diseases were excluded in five studies (20, 22–24, 27). Of these, three studies also excluded patients with infectious diseases at the time of sampling to confound for potential impact on Treg populations (22, 23, 27). One study also listed the withdrawal criteria for women with inflammation or infection at the time of sampling (24).

Immunophenotyping of Tregs in all studies was done using flow cytometry. The exact markers and methodology are listed in **Table 1**. Most studies took samples at one time point only, while two studies followed the women taking samples postpartum (23, 24). Only samples taken within pregnancy were included in the meta-analysis. Lobo was the only study to measure multiple Treg markers (22). The exact markers and individual study results can be seen in Tables 2.1 (Appendix 3) and 2.2 (Appendix 5). All studies were matched for gestational age. Two studies were age- and weight-matched with controls (24, 27).

**Table 1:** Study characteristics (includes setting, population, diagnostic criteria, number of participants in each group divided by management, inclusion and exclusion criteria, overall findings, and specified Treg markers) of the included studies.

Paper	Country and sample source	Study design	GDM diagnostic criteria	Trimester at sample	GDM (*N*)			Control (*N*)	Inclusion criteria	Exclusion criteria	Tregs marker and method of retrieval	Overall conclusion (overall population of Tregs)	Additional findings
					D	I/M	Total	Total					
Schober 2014 (21)	Italy; ultrasound and delivery room section of the hospital	Case–control	IADPSG 2-h 75-g OGTT	Third	21	40	61	64	No specific criteria	• N/A	CD4^+^CD127^low+/−^CD25^+^FOXP3^+^ Cells were analyzed using a FACSCanto cytometer	No significant difference	Treg subsets and suppressive activity of Tregs pool measured.
Lobo 2018 (22)	Brazil; antenatal clinic between 2014 and 2016	Case–control	IADPSG 2-h 75-g OGTT	Third	–	–	31	27	• Pre-pregnancy BMI ≥25 kg/m^2^ • GA 28–38 weeks at recruitment • Singleton pregnancy • Live fetus	• Infection • Systemic disease • Other obstetric disorders	1) CD3^+^CD4^+^CD25^+^ 2) CD3^+^CD4^+^CD25^+^FOXP3^+^ 3) CD3^+^CD4^+^CD25^+^FOXP3^+^CD127^−^ Cells were evaluated in the BD Aria III cytometer. To confirm the presence of Treg populations, cells were stained with specific markers and the samples were immunophenotyped to assess purity (98%).	No significant difference	Cytokine and chemokine production in Tregs and NK cells
Sheu 2018 (23)	Australia; women with GDM were recruited from the GDM clinic and the control group was recruited from the antenatal clinic of the Royal Hospital for Women, Sydney between May 2013 and October 2015	Case–control	ADIPS 1-h 50 g OGCT; from January 2015: IADPSG 2-h 75-g OGTT	Third	–	–	55	65	• No specific criteria • No exclusion of patients with GDM in previous pregnancies	• Multiple pregnancy • Autoimmune disease • Type 1 or type 2 diabetes • Hypertensive disorders (pre-existing/new hypertension) • Immunosuppressive agents (e.g., glucocorticoids) • IM steroids • Respiratory infection • Inflammatory illness	CD4^+^CD25^+^CD127^−^ Treg populations were isolated using a five-laser Fortessa flow cytometer	No significant difference	Secondary sample 7 weeks postpartum T helper cell populations and the ratio of Th to Tregs measured.
Yang 2018 (20)	China; hospital	Cohort	Carpenter and Coustan criteria (1982) 3-h OGTT	First	–	–	21	34	• Newly pregnant women in the first trimester • BMI ≥25 kg/m^2^ • Chinese	• GDM in immediate family • Pre-existing type 1 diabetes • Pre-existing type 2 diabetes • Medication at sample collection	CD4^+^CD25^+^FOXP3^+^ The peripheral blood mononuclear cell (PBMC) layer was frozen and thawed when ready to use. Cells were identified using a FACSCanto II.	Significantly lower in GDM	Serum IL-6, IL-10, TNF-α, and TGF-beta were measured.
Sifnaios 2019 (24)	Greece; outpatient clinic	Case–control	IADPSG 2-h 75-g OGTT	Third	26	0	26	23	• Adult • Caucasian • No other comorbidities • Primigravida	• TD2M/overt TD2M • Smoking • Hx/presence of systemic/inflammatory disease • hX of prescribed medications in the last 1 year Insulin treatment of GDM•	CD3^+^CD4^+^IL-10^+^ as expressed by Tregs Samples were analyzed on a BD FACSCanto II flow cytometer	Significantly higher in GDM	Secondary sample 6 months postpartum. Th1 and Th2 responses measured. Measurement of all markers during post-pregnancy as well
Fagninou 2020 (27)	Benin; specialist clinicians enrolled participants from three national hospitals	Cross-sectional case–control	IADPSG 2-h 75-G OGTT	Third			15	25		• Clinical coronary artery disease • Renal disease • Hepatic diseases • Clinical signs of infectious disease	CD4+CD25+CD127-FoxP3+ Stained cells were acquired using a FACSCanto II flow cytometer	No significant difference	Serum IL-10 and Th1 and Th2 ratio measured. NK cells and monocytes also measured
Zhao 2020 (25)	China; pregnant women were recruited from the Yantai Yuhuangding Hospital between Jan 2019 and October 2019	Prospective cohort	IADPSG 2-h 75-g OGTT	Third	16	12	28	28			CD3^+^CD4^+^CD25^+^CD127^−^ Cells were analyzed on a FACSCanto II flow cytometer	Tregs lower in GDM	Treg proportions compared in CD8^+^ cells and in cord and retro-placental blood
Wang 2022 (26)	China; pregnant women were recruited from the Yantai Yuhuangding Hospital between January 2019 and October 2019	Prospective cohort	IADPSG 2-h 75-g OGTT	Second and third trimesters			45 (17 = 2^nd^.28 T = 3^rd^ T)	104 (28 in the first trimester, 43 in the second trimester, 33 in the third trimester			CD3^+^CD4^+^CD25^+^CD127^−^ Cells were analyzed on a FACSCanto II flow cytometer	Tregs lower in GDM	Non-pregnant women and healthy pregnant controls in all trimesters. CD8^+^ Treg populations also measured

The GDM group is split into D for diet-controlled, I/M for insulin/medication-controlled, and total.

Two studies were matched for weight only (20, 22). Ethnicity was documented in four studies (22, 23), which was controlled as a confounding factor between groups in two studies (20, 24). Tregs were measured in six studies in the third trimester (21–25, 27) and in one study each in the first (20) and second (26) trimesters. There were two studies on overweight women with a BMI ≥25 kg/m^2^ (20, 22).

**Table 1** shows the individual study characteristics in detail, including the diagnostic criteria and management. **Table 2** highlights the maternal characteristics as published by the studies. **Table 3** shows all data relevant to Tregs. This includes additional data on subpopulations and other immune markers. The measured child characteristics are listed in Appendix 6. The detailed criteria and management of GDM by individual studies are listed in **Tables 1**, **2** (21, 24, 25). Only three studies defined the management status of the patients. Of these, only one excluded patients managed with insulin (24), while the remaining two documented those diet-controlled and insulin-controlled but did not differentiate between them when sampling Treg populations. This is further expanded in Appendix 7.

**Table 2:** Maternal characteristics.

Paper	GA at sample collection (weeks)		Age (years)		Weight/BMI		Ethnicity		Fasting glucose	
	GDM	Control	GDM	Control	GDM	Control	GDM	Control	GDM	Control
Schober 2014 (21) [Median (range)]	Diet: 39 (24–41) Insulin: 36 (24–42)	37 (24–41)	Diet: 32 (25–43) Insulin: 34 (22–43)	31 (21–44)	Pre-pregnancy: diet: 67 (45–126), insulin: 89 (54–153) kg Post-pregnancy: diet 83 (58–136), insulin 100 (54–160) kg	Pre-pregnancy: 66 (50–117) kg Post-pregnancy 81 (59–128) kg	Not specified	Not specified	Not specified	Not specified
Lobo 2018 (22) (Mean ± SD)	34.14 ± 1.99	33.51 ± 2.99	34.74 ± 4.45	28.33 ± 5.52	Pre-pregnancy: 29.61 ± 4.49 kg/m^2^	Pre-pregnancy: 29.96 ± 4.28 kg/m^2^	35.48% Caucasian Mixed: 48.39% Black: 16.13%	White: 40.74% Mixed: 48.15% Black: 11.11%	91.20 ± 5.49 mg/dL	85.04 ± 5.62 mg/dL
Sheu 2018 (23) (mean ± SD unless otherwise stated)	36–38 (range)	36–38 (range)	33.9 ± 3.6	33.2 ± 4.5	25.1 ± 6.8 kg/m^2^	25.1 ± 5.5 kg/m^2^	49.1% Caucasian, 50.9% non-Caucasian	72.3% Caucasian, 27.7% non-Caucasian	4.8 ± 0.6 mmol/L	4.3 ± 0.4 mmol/L
Yang 2018 (20) (Mean ± SD)	10.8 ± 0.9	10.3 ± 1.1	32.1 ± 5.1	32.5 ± 4.2	31.7 ± 3.1 kg/m^2^	31.6 ± 3.7	Chinese	Chinese	5.2 ± 0.4 mg/dL	5.2 ± 0.4 mg/dL
Sifnaios 2019 (24)	28–34 (range)	Range 28–34	34.0 ± 3.7	31.6 ± 5.2	29.5 ± 11.8 kg/m^2^	26.2 ± 8.5 kg/m^2^	Caucasian	Caucasian	Not specified	Not specified
Fagninou 2020 (27)	28–35 (range)	28–32	30.6 ± 3.04	29.19 ± 3.94	Not specified	Not specified	Not specified	Not specified	1.23 ± 0.05 g/L	0.8 ± 0.03 g/L
Zhao 2020 (25) (Mean ± SEM)	39.58 ± 0.32	39.59 ± 0.18	27.45 ± 1.25	26.74 ± 1.18	Not specified	Not specified	Not specified	Not specified	85.76 ± 1.39 mg/dL	89.48 ± 0.76 mg/dL
Wang 2022 (26) (Mean ± SEM)	26.5 ± 0.1 (second trimester) 39.4 ± 0.4 (third trimester)	26.2 ± 0.2 (second trimester) 39.5 ± 0.1 (third trimester)	26.8 ± 1.7 (second trimester) 26.4 ± 0.3 (third trimester)	27.3 ± 1.8 (second trimester) 27.4 ± 1.2 (third trimester)	23.1 ± 1.3 kg/m^2^ (second trimester) 26.1 ± 1.6 kg/m^2^ (third trimester)	22.6 ± 1.9 kg/m^2^ (second trimester) 25.7 ± 1.7 kg/m^2^ (third trimester)	Not specified	Not specified	4.9 ± 0.7 mmol/L (second trimester) 4.9 ± 0.3 mmol/L (third trimester)	4.6 ± 0.8 mmol/L (second trimester) 4.6 ± 0.3 mmol/L (third trimester)

**Table 3:** Treg populations measured in women with GDM and pregnant controls against the individual Treg markers used in the studies (mean and standard deviation).

Paper	Treg marker	Units used in the paper	Mean ± SD		P-value	Other immune markers measured	Data extracted from the graph
			GDM	Control			
Schober 2014 (21)	CD4^+^CD127^low+/−^CD25^+^FOXP3^+^	Median (range)	4.9428±1.11516942	5.2202 ± 1.1127	*p* > 0.05	Treg subsets: • Naive CD45RA^+^ Tregs: % decreased significantly in both diet-adjusted and insulin-controlled GDM • HLA-DR^−^CD45RA^−^ memory Tregs (DR-Tregs): % increased significantly in diet-controlled GDM • HLA-DR^low+^CD45RA^−^: % increased significantly in insulin-controlled GDM • HLA-DR^high+^CD45RA^−^ memory Tregs (DR^low+^ and DR^high+^ Tregs): increased significantly in insulin-controlled GDM	No
Lobo 2018 (22)	1) CD3^+^CD4^+^CD25^+^ 2) CD3^+^CD4^+^CD25^+^FOXP3^+^ 3) CD3^+^CD4^+^CD25^+^FOXP3^+^CD127^−^	Mean ± SD	1) 0.93953 ± 0.86512 2) 0.37209 ± 0.38139 3) 0.02046 ± 0.30512	1) 0.9116 ± 0.8744 2) 0.45581 ± 0.4093 3) 0.3068 ± 0.2884	*p* > 0.05	• TCD4^+^: no significant difference • TCD3^+^CD4^+^CD25^+^: no significant difference • CD25^bright^: there was a significantly lower percentage in GDM • FOXP3^high^: % was significantly lower in GDM • NK cells: no significant difference • CD56^dim^: there was a higher frequency in GDM • NKT cells: no significant difference • TNF-α production by Treg cells: higher in GDM • No significant difference was observed in other cytokines and chemokines produced by Treg and NK cells (including IL-10)	Yes
Sheu 2018 (23)	CD4^+^CD25^+^CD127^−^	Median (IQR)	4.3391 ± 2.3828	4.853 ± 0.887	*p* > 0.05	• Proinflammatory cells (Th17, Th17.1, Th1): women with GDM had a higher % of Th17 cells. GDM patients also had higher proinflammatory ratios (Th17:Treg, Th17.1:Treg, and Th1:Treg). These percentages and ratios declined significantly after delivery in GDM patients but not in controls	Yes
Yang 2018 (20)	CD4^+^CD25^+^FOXP3^+^	Mean ± SD	0.95 ± 0.38	1.28 ± 0.40	*p* = 0.0156 (lower in GDM)	• CD4+CD25+T cells: reduced in GDM • Serum IL-6: increased in GDM • IL-10: significantly lower in GDM • TNF-α: increased in GDM • TGF-beta: significantly lower in GDM	Yes
Sifnaios 2019 (24)	CD3^+^CD4^+^IL-10^+^	Median (IQR)	0.2 ± 0.1569	0.0358 ± 0.079	*p* < 0.001 (IL-10 expression higher in GDM)	• CD3+CD4+IFN-y (Th1 response): no significant difference • proportion of IL-13 expressing CD3+CD4+IL-13+ (Th2 response): significantly higher in GDM. This was reversed 6 months post-delivery so it was significantly higher in the control • CD3^+^CD4^+^IL-17^+^ (Th17 response): significantly higher in GDM. Six months post-delivery, there was no significant difference.	No
Fagninou 2020 (27)	CD4^+^CD25^+^CD127^−^FoxP3^+^	Median and IQR	5.6956 ± 2.4046	5.0482 ± 3.907	*p* > 0.05	Serum IL-10 levels were found to be lower in GDM, while total CD4^+^ cell frequencies were higher in women with GDM.	Yes
Zhao 2020 (25)	CD3^+^CD4^+^CD25^+^CD127^−^	Median (IQR%)	3.6507 ± 1.5233	7.3448 ± 2.2107	P < 0.01 (lower in GDM)	No significant difference in CD3^+^, CD4^+^, and CD8^+^ T cells found between the groups	Yes
Wang 2022 (26)	CD3^+^CD4^+^CD25^+^CD127^−^	Mean ± SEM	2nd trimester = 4.87 ± 1.4843183rd trimester GDM = 4.25 ± 2.063686	2nd trimester = 7.67 ± 0.9180413933rd trimester = 6.38 ± 2.92972695	P < 0.05 (2nd) P < 0.01 (3rd) Lower in GDM	CD8^+^ Tregs also measured: CD8^+^ Tregs higher in GDM	No

Published units are defined in the column; therefore, studies that were not published as mean ± SD were converted accordingly. A p-value >0.05 denotes no significant statistical value. Studies where data were extracted from graphical presentations are also documented as “yes” under the column “data extracted from graphs.” Additional findings of individual studies are mentioned under “other markers used.” Of note are the measured Treg subpopulations by Schober et al. (2014) (21) and measurement of increased proinflammatory populations by Sheu et al. (2018) (23) and postpartum by Sifnaios et al. (2019) (24).

Overall, the study quality assessed, using the modified NOS scale, was considered fair with a medium/low risk of bias. The full assessment and the scores awarded are available in Appendix 5.

### Analysis

The third-trimester data from Wang et al. (26) were excluded from the quantitative analysis due to the high probability that these data were already published by Yang et al. (25). The second-trimester data were included.

**Figure 2:**
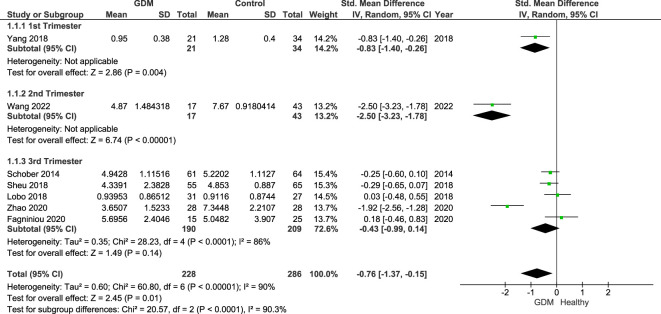
Forest plot of the SMD of women with GDM vs. control subgrouped by trimesters. The figure shows that overall Treg populations are found to be lower in women with GDM. Individually by trimester, significant differences were shown in the studies of women in the first (*p* = 0.004; SDM = −0.83; CI: −1.40, −0.26) and second trimesters (*p* = 0.004; SDM = −2.50; CI: −3.23, −1.78) reflective of the published individual study findings. However, when comparing studies in the third trimester only, the overall SDM was −0.43 (95% CI: −0.99, 0.14; *I* ^2^ = 84%). As the CI crosses the null value, there is no overall significance. In the third trimester, only one study reported a significantly lower number of Tregs in women with GDM with the other four reporting and showing a non-significant difference.

**Figure 2** shows the summary forest plot of the analysis of the seven studies across all three trimesters (GDM = 228, control = 286). These were subgrouped by trimester: one study in trimester 1, one study in trimester 2, and five studies in trimester 3. Results show that Treg populations are significantly lower in women with GDM with an SMD of −0.76 (95% CI −1.37, −0.15 and *I* ^2^ = 90%; *p* = 0.01). A large heterogeneity between the studies as highlighted by the *I* ^2^ values was unexplained by the sensitivity analysis.

**Figure 3:**
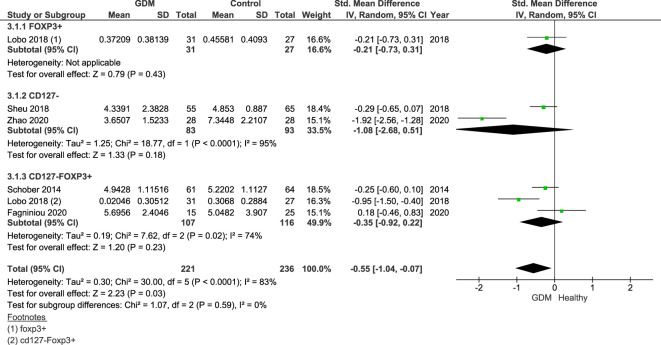
Forest plot of GDM vs. control subgrouped by the Treg markers Foxp3^+^, CD127^−^, and CD127^−^Foxp3^+^. The figure shows that overall Treg populations are found to be lower in women with GDM.

Additional analysis, subgrouped by Treg markers, was done on the five studies measuring Tregs in the third trimester only (**Figure 3**). The groups of markers were as follows: CD4^+^FoxP3^+^ (22) (women with GDM = 31, control = 27), CD4^+^CD127^−^ (23, 25, 26) (GDM = 83, control = 93), and CD4^+^CD127^−^FoxP3^+^ (21, 22, 27) (women with GDM = 107, control = 116). Lobo et al. measured the populations of both CD4^+^FoxP3^+^ and CD4^+^CD127^−^FoxP3^+^, and therefore, the published results of both populations were included in the subgroup analysis.

Overall, the SDM of the Treg populations specific to Treg markers was −0.55 (95% CI = −1.04, −0.07; *I* ^2^ = 83%). This shows lower proportions of Tregs across all markers in the third trimester. Individually, all three markers cross the null value: Foxp3^+^ SDM = −0.21 (95% CI = −0.73, 0.31), CD127^−^ SMD = −1.08 (95% CI = −2.68, 0.51; *I* ^2^ = 95%), and CD127^−^Foxp3^+^ SDM = −0.35 (95% CI = −0.92, 0.22; *I* ^2^ = 74%).

**Figure 4:**
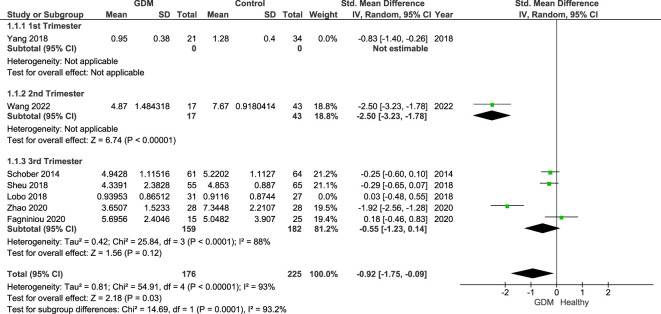
Forest plot of women with GDM vs. control done for sensitivity analysis excluding studies that included overweight/obese women only. The figure shows that overall Treg populations remain lower in women with GDM with CI below the null value.

**Figure 4** shows the sensitivity analysis excluding studies (20, 22) that only included overweight/obese women, as obesity has previously been linked to a dysregulation in Treg populations in the “adipose tissue.” The results remain statistically significant (*p* = 0.03) of the Treg populations of women with GDM compared with healthy pregnant controls with an SMD of −0.92 (95% CI: −1.75, −0.09; *I* ^2 = ^93%).

Additional findings from individual studies that measured additional parameters related to Treg populations and expressive activity as well as other immune markers are listed in Appendix 8. A concise overview is listed in **Table 3**.

## Discussion

The results of the meta-analysis suggest that GDM is associated with lower levels of Tregs circulating in the peripheral blood of affected mothers when compared with healthy pregnant controls. Likewise, the pooled analysis of only third-trimester samples, by specific Treg cell markers Foxp3^+^, CD127^−^, and CD127^−^Foxp3^+^, reflected the main findings (**Figure 2**). These findings reflect previous evidence of dysregulation of Tregs in obstetric and non-obstetric insulin-dependent complications (14, 16, 35).

However, subgroup analysis of the third-trimester studies (when using only the main Treg marker) was non-significant. This needs to be further investigated due to limited sample sizes, and possible explanations for this result may be attributed to the dynamic nature of the immune response during normal pregnancy and the differences in Treg markers and subpopulations.

This suggests that specific Treg subpopulations may be implicated in GDM even if the overall number of Treg cells does not reflect the complex nature of studying Tregs. This theory is further implied by the findings in some of the individual studies. Despite no significant differences being found in overall Treg cell populations, significant differences were found in the suppressive ability of Tregs through expression of specific markers such as IL-10 or in gating for naive and memory Treg subsets (23, 24).

Treg cell subpopulations and the expression of specific markers relative to their suppressive ability are complex subjects that require further research, especially in the context of GDM; thus, these cannot be expanded in this paper.

With regard to pregnancy, early studies into immune regulation embraced pregnancy as a “Th-2” or anti-inflammatory state, postulating that a shift in cytokine production would result in abortion or significant pregnancy complications. However, this theory was argued against, and subsequent research paints a complex picture whereby the immune response is seen to alter throughout pregnancy (36). The initial studies evaluated pregnancy as a single event, where in reality, every stage has different requirements on the maternal system. Broadly speaking, it is understood that the first, early second, and late third trimesters require a proinflammatory environment for implantation and later delivery, while the second trimester, a period of rapid fetal growth and development, is an anti-inflammatory stage (37).

It has also been established that Tregs increase during normal pregnancy and seem to play a pivotal role in the mechanism of successful pregnancy. However, Treg numbers and subpopulations vary across women and within individuals throughout pregnancy, with a decline typically observed in the third trimester (13, 38, 39).

The pattern of changes in the percentage of circulating Tregs is suggested to follow this trajectory: an increase in the first trimester as opposed to non-pregnant individuals, peaking in the second trimester, and subsequently declining in the third trimester (39). Furthermore, a significant decrease in Treg levels has been observed by several studies at the time of delivery (38–40), which may also account for the non-significant differences between the affected and healthy groups shown in the analysis in the third trimester.

Thus, we must consider the potential impact of possible fluctuations in Treg populations as pregnancy progresses and the association these may have with diseases such as GDM.

Both the dynamic nature of the immune response in normal pregnancy and GDM and the complexity in understanding and measuring Treg populations, function, and expression reiterate the requirement for further research in this area.

### Strengths

This review fills a critical gap in the literature by being the first to gather all the evidence in this area. Our findings can therefore be used to aid future research regarding disease pathophysiology with a potential impact on clinical practice. We used the Cochrane Handbook as a guide when planning and conducting this review and adhered to a clear definition of search strategy and inclusion and exclusion criteria.

### Limitations

This study is subject to several limitations primarily stemming from the scarcity of published studies with small cohort sizes and high heterogeneity among the trials. Additionally, the included studies have examined different Treg cell markers. This is a valid current practice with results being deemed comparable for analysis; however, to further increase understanding, there is a necessity to have a consensus on the adoption of universal markers (30).

Likewise, recent evidence shows that Tregs have subpopulations based on the expression of cytokines and they can also be classified into naive and effector Tregs (30, 41, 42), but most studies did not differentiate between these. The role of Treg plasticity is also of interest. Tregs are suggested to have functional plasticity whereby they maintain the expression of Foxp3 and the required chemokine receptors yet have the capacity to acquire a different phenotype (e.g., Th1, Th2, and Th17 Tregs) in response to environmental cues and anatomical location (11).

Due to data limitations, we were unable to perform subgroup analysis by severity of disease (judged by glycemic status or management strategy: diet-controlled vs. medication). Previous studies have found a significantly lower proportion of Tregs in insulin-controlled TD2M compared with diet-controlled (43); therefore, a future comparison between Tregs depending on the severity of GDM is desired.

Additionally, the data for women in their first and second trimesters were notably scarce, most likely due to the limitations of current diagnostic methods. The ethnicity of participants was also overlooked in many studies. As it is established that some ethnic groups are at a higher risk of GDM than others (2), pathophysiology may also differ. Consequently, this demographic information may be critical in translating research findings into clinical practice. We hope our study highlights and encourages the need for further research in these areas.

### Clinical and research implications

This review suggests that Treg populations may be significantly lower in women with GDM; however, the mechanism of this association or causal relationship is undetermined. Likewise, literature specific to studying Tregs in GDM is also limited. Thus, we need a better understanding of the role of Tregs in the pathophysiology of GDM as well as considerations of the impact of factors associated with GDM such as obesity, gestational age, and maternal ethnicity on Treg populations. We also need to consider the impact of Treg activity and the role of Treg plasticity in these diseases (11).

**Figure 5:**
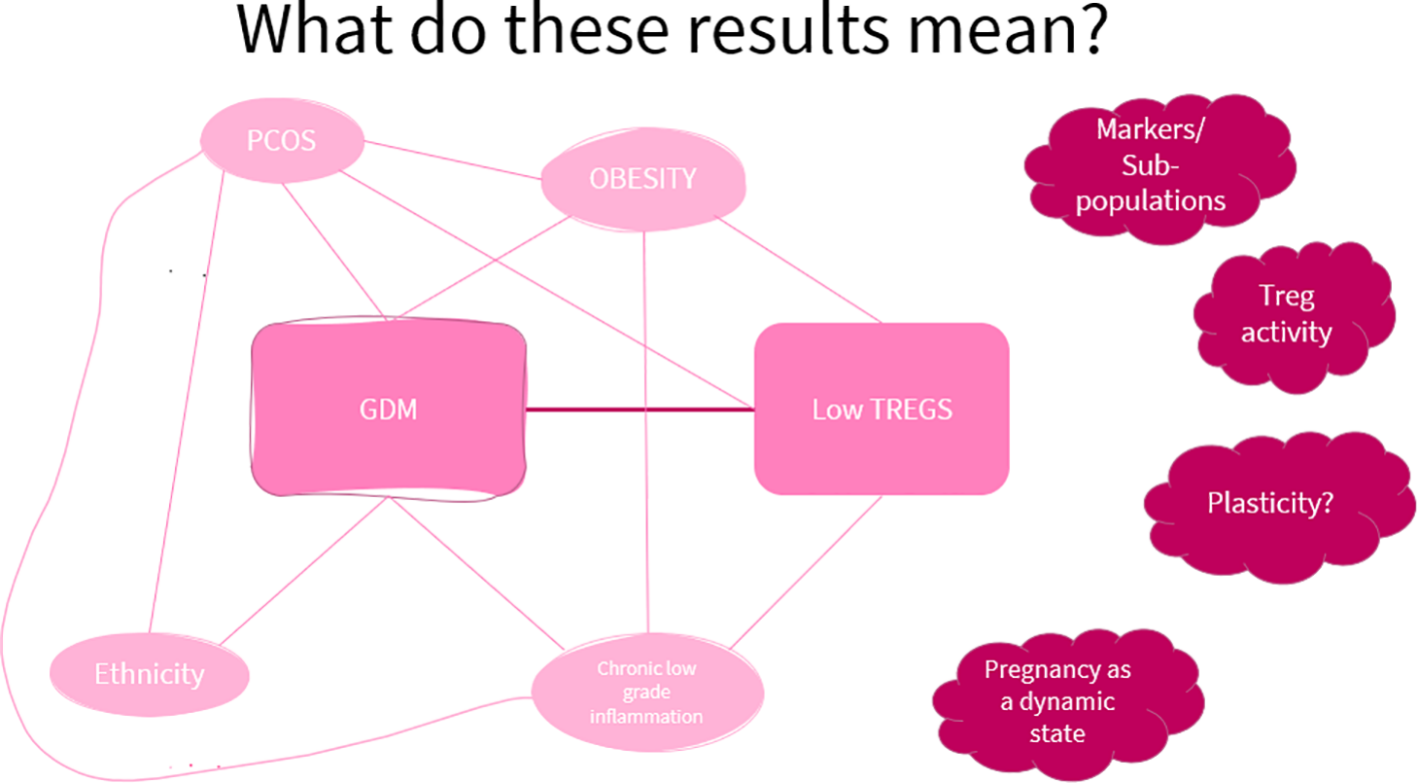
A visual aid of the factors showing a link between GDM and low Treg levels. This shows that though there may be an association between low Treg levels and GDM, current evidence does not allow a conclusion on the causal relationship between the two.

**Figure 5** illustrates the factors that are common between GDM and low Treg levels.

The use of Tregs as a diagnostic marker for GDM is unlikely due to lack of disease specificity as highlighted by the range of diseases associated with Treg pathology. However, there may be future potential for the use of Tregs as an early prognostic marker for obstetric complications in comparison to current diagnostic measures in later pregnancy, leading to better diagnosis and management, if we can determine appropriate threshold levels.

**Figure 6:**
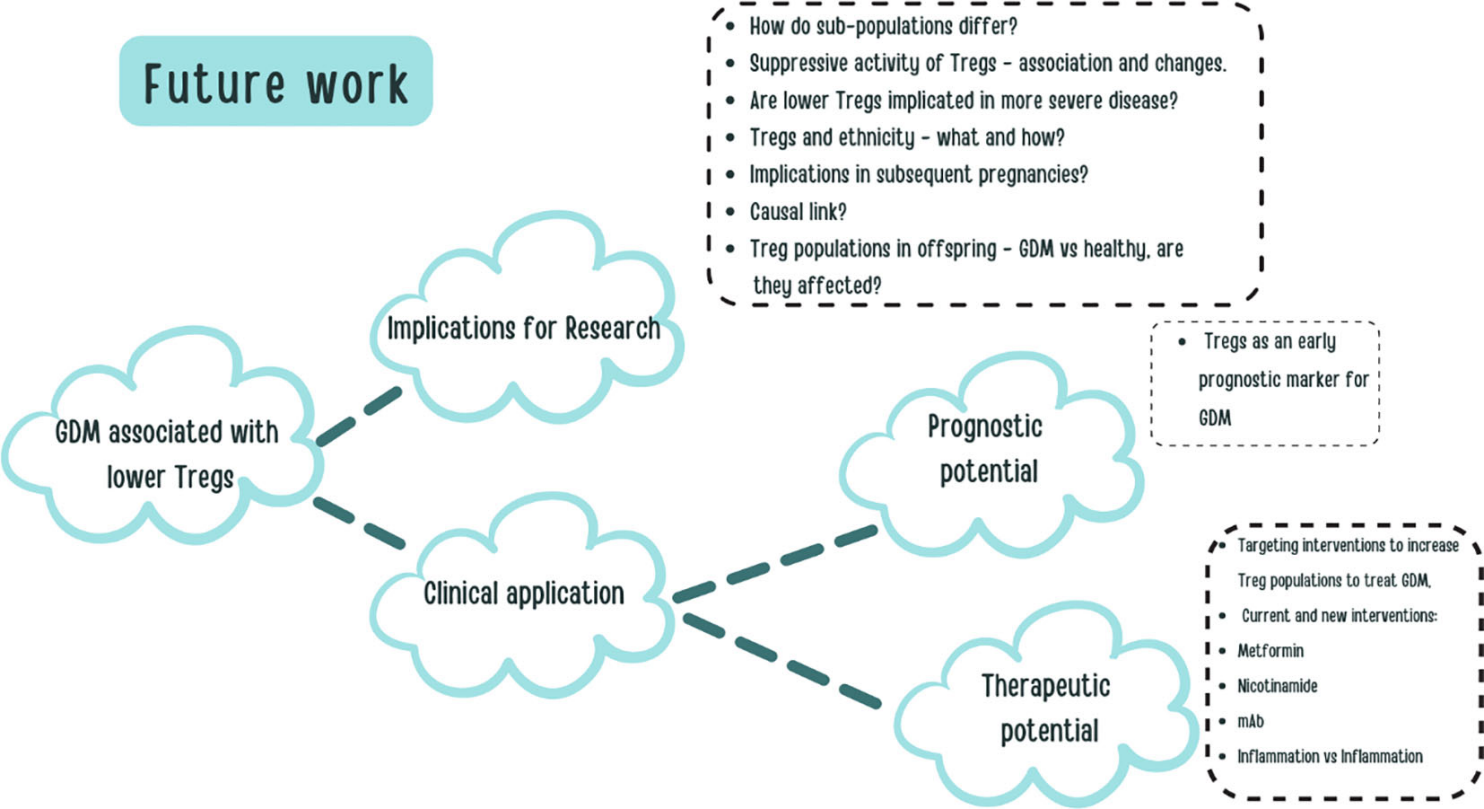
A visual aid constructed to explore some of the potential future work and clinical applications based on our research.

**Figure 6** shows a diagram of the implications of the results for future work.

## Conclusion

The study has demonstrated that GDM is associated with lower maternal blood Treg numbers (44–47). However, this finding does not imply causation, and the precise mechanisms by which Tregs may mediate GDM remain unclear. Furthermore, although the authors of individual studies included in the meta-analysis acknowledged and accounted for some factors, there is a possibility that these findings are related to other/unknown underlying mechanisms and associated risk factors which may lead to lower Tregs in GDM (e.g., obesity) or physiological mechanisms associated with the disease such as the associated state of chronic low-grade inflammation (35, 43). Nevertheless, our findings add to the evidence base implicating a dysregulation of Treg cell number in disease pathology further incentivizing research into Tregs in GDM.

## Data availability statement

The original contributions presented in the study are included in the article/supplementary material. Further inquiries can be directed to the corresponding author.

## Author contributions

PS, HA, and TP conceptualized the topic and structure of the systematic review. PS, HA, and TP drafted and revised the manuscript. NM, AE, GL, TT, KHN, and PS provided expert opinions and edited and approved the final manuscript. All authors contributed to the article and approved the submitted version.
