# Supplementary material for: Regulatory T cells in the peripheral blood of women with gestational diabetes: a systematic review and meta-analysis
Source: Front Immunol. 2023 Dec 4;14:1226617. doi: 10.3389/fimmu.2023.1226617 (PMC10726109; doi:10.3389/fimmu.2023.1226617)
Supplement: Supplementary file 1 [file DataSheet_1.docx]

**Appendix 1**

The search words were as follows: ‘Tregs’ OR ‘regulatory T-cells’ OR ‘T cells’ OR ‘T lymphocytes’ OR ‘CD4+’ OR ‘CD12’ OR ‘FOXP3+’ OR ‘thymus cells’ OR ‘HLA-DR’ AND ‘gestational diabetes’.

**Appendix 2: Formulae for combining groups**

**
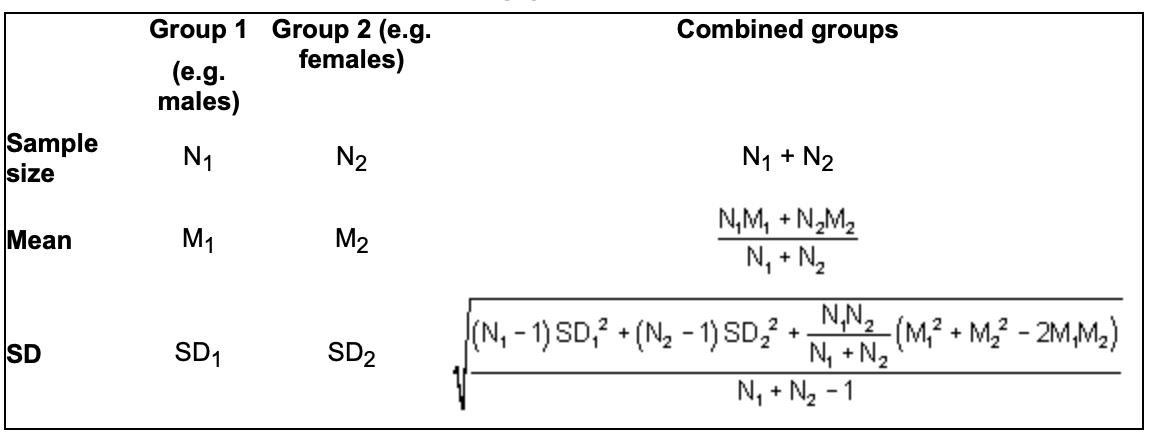
**

Extracted from https://handbook-5-1.cochrane.org/chapter_7/table_7_7_a_formulae_for_combining_groups.htm

**Appendix 3: Modified Newcastle-Ottawa scale**

To allow both reviewers to assess this consistently, criteria within the three domains were devised specifically for the review (table 2.1). Blinding in cohort studies was difficult and perhaps not as necessary as other studies due to the nature of the studies, i.e. testing for Treg populations in a lab at a given point in pregnancy. For cohort studies, an initial discussion of criteria led to the conclusion that blinding was not necessary and therefore a star should be awarded to all, however the subsequent conclusion reached was that studies could blind by ensuring those testing samples and carrying out flow-cytometry are blinded to the GDM status of women – this was to be made clear in the methods section or would otherwise be deemed as not blinded. Alternatively, the scale allows for a star to be awarded if the confirmation of outcome is done through secure records; this was therefore defined as GDM diagnosis using an accredited test for both groups.

Likewise, follow up samples could be taken at subsequent points in pregnancy/ post-partum or follow up of glucose-tolerance status could be achieved and therefore stars awarded accordingly. Loss of response rate was not applicable for studies that only looked at one-time point and therefore stars were awarded regardless. Studies that did not control for potential confounders that could impact Tregs profile (defined as GA, the weight of patients and disease/infection that could impact lymphocyte populations) were awarded lower scores.

As per the Newcastle-Ottawa scale, hospital settings for participant recruitment were marked down reflecting a lower representation of the community. However, emphasis was placed on recruitment criteria specific to the reason for being in hospitals/ departments, specifically recruitment from routine appointments in antenatal clinics (which were awarded a star). This was likewise for comparison with control and therefore studies where controls were recruited from the same departments as women with GDM, were awarded a star for selecting controls. Following independent classification, the reviewers held a discussion to compare results and a consensus was reached.

*Table 2.1: modified Newcastle-Ottawa scale*

*Table 2.1 shows the criteria established between reviewers for Newcastle-Ottawa Scale defined by study design as used to assess study quality by the reviewers*

|  | Cohort | Case control |
| --- | --- | --- |
| Selection (1-4) | 1. Independent validation defined as OGTT/OGCT 2. Community representative/ routine antenatal appointments 3. Should be same as participants according to the criteria of the study. 4. Do not have to define previous GDM status | |
| Comparability | 1. Gestational Age 2. Confounding for weight/BMI, infections, autoimmune disease, medications etc | |
| Exposure/outcome | 1. No blinding required; outcome defined as GDM 2. Sample method and glucose screen 3. Follow up > 1 month | 1. Sample method and glucose screen 2. As above 3. Loss of follow up |
| Comments | Comment of strengths and weakness of the study which contributed to awarding of stars. | |

**Appendix 4:**

205 studies were excluded after abstract screening for not meeting the inclusion criteria listed below. The exclusion criteria specifies reasons for the exclusion of these studies. 14 texts were identified for full text screening, 9 of which were included for the reasons listed in the table below.

Inclusion criteria:

Inclusion criteria were original human studies (all study designs) measuring Treg levels in the peripheral blood of pregnant women with GDM, published in the English language.

Exclusion criteria

Unpublished literature, abstracts, literature reviews, animal studies, studies published in another language without translation into English, studies of participants with previous immune dysregulation (e.g. women with HIV), samples taken from cord blood or decidua and studies with ambiguity in methodology identifying Tregs.

**Studies not included after being identified for full text screening:**

| Study identified for full-text screening but subsequently excluded | Title | Reason |
| --- | --- | --- |
| Lapolla, et al. 1999 | A study on lymphocyte subpopulation in diabetic mothers at delivery and in their newborn | Language – study in Italian |
| Friebe-Hoffmann, et al. 2017 | Peripheral Immunological Cells in Pregnant Women and their Change during Diabetes | Inability to access full text despite effort from both reviewers and supervisor PS. |
| Studies excluded after full-text screening |  |  |
| Mario, et al 1987 | Immunology in diabetic pregnancy: activated T cells in diabetic mothers and neonates | No mention of Tregs |
| Lapolla et al, 2005 | Lymphocyte subsets and cytokines in women with gestational diabetes mellitus and their newborn. | Tregs not measured |
| Mahmoud, et al. 2005 | Lymphocyte sub-populations in gestational diabetes. | Tregs not measured |
| Mahmoud, et al. 2006 | Butyrylcholinesterase activity in gestational diabetes: correlation with lymphocyte subpopulations in peripheral blood | Treg populations were not differentiated from overall CD4+CD25+ T helper cell populations and the presumption was made that a significant proportion are Tregs but not all. Therefore, this study was not included in the synthesis. |
| Mahmoud et al, 2012 | Trace elements and cell-mediated immunity in gestational and pre-gestational diabetes mellitus at third trimester of pregnancy | Tregs not measured |
| Pendeloski et al, 2015 | Immunoregulatory  molecules in  patients with  gestational diabetes  mellitus. | Tregs not measured |
| Fagundes et al, 2015 | Changes in T-cell phenotype and  cytokines profile in maternal blood, cord blood and colostrum of diabetic mothers | Tregs not measured |

**Appendix 5**

Table 2.2 Newcastle-Ottawa scale quality assessment by study

| Study  Case-control | Selection (4) | Comparability (2) | Exposure/  Outcome (3) | Total | Comments |
| --- | --- | --- | --- | --- | --- |
| Schober et al, 2014 | **2** | **1** | **3** | **6 fair** | Confound for GDM management by splitting into insulin and diet-controlled but despite measuring weight of women, do not confound for it. |
| Lobo et al, 2018 | **4** | **2** | **3** | **9 - Good** | Confound for weight, infection, and other co-morbidities but no follow up.  Overweight women recruited from antenatal clinic (GMD and control). Exclusion criteria also accounted for other potential confounding factors such as infections. |
| Sheu et al, 2018 | **2** | **1** | **3** | **6 – Fair** | Extensive criteria of potential confounding factors of control but do not mention controlling for weight between groups. Control of similar factors in women with GDM is also unclear. |
| Sifnaios et al, 2019 | **9** | **2** | **3** | **9 - good** | Overall good study with extensive inclusion criteria specific to factors that may impact only looked at Caucasian women and controls matched for age and BMI. Follow up 6 months post birth |
| Fanginiou et al, 2020 | **2** | **2** | **3** | **7 – good** | Tertiary hospital recruitment from 3 centres as per study criteria however the criteria is not made sufficiently clear. Acknowledgment that  age and BMI were matched when recruiting. |
| Cohort |  |  |  |  |  |
| Yang et al 2018 | **3** | **2** | **3** | **8 – good** | Weight matched newly pregnant women tested and followed into pregnancy to see if they develop GDM. All subjects ethnically Chinese reflective of population rather than exclusion. |
| Zhao et al, 2020 | **3** | **1** | **2** | **6 – fair** | Women coming in for delivery. No follow up specified |
| Wang et al 2022 | **2** | **1** | **2** | **5 - fair** | Different cohorts of women recruited per trimester, extensive control recruitment of healthy pregnancy and non-pregnant women. However, does not define where GDM women were recruited from. No follow up. |

Table 2.2 shows the scores and comments relevant to scores for each study based on the modified Newcastle-Ottawa scale

**Appendix 6**

Table 2.3 Offspring characteristics

| Paper | Birth weight (g) | | Birth Length (cm) | | Atopic Profile n (%) | |
| --- | --- | --- | --- | --- | --- | --- |
|  | **GDM** | **Control** | **GDM** | **Control** | **GDM** | **Control** |
| Schober 2014 | N/A | N/A | N/A | N/A | N/A | N/A |
| Lobo 2018 | N/A | N/A | N/A | N/A | N/A | N/A |
| Sheu 2018  (mean ± SD) | 3316 ± 376 | 3540 ± 399 | 50.8 ± 2.9 | 51.9 ± 2.5 | 8 (30.8) | 0 (0) |
| Yang 2018 | N/A | N/A | N/A | N/A | N/A | N/A |
| Sifnaios 2019  median (IQR) | 3100 (2600 -3500) | 3200 (3000 -3300) | 50 (48-51) | 50.5 (49.3-51.5) | N/A | N/A |
| Fangninou 2020 | N/A | N/A | N/A | N/A | N/A | N/A |
| Zhao 2020  (mean ± SEM | 3634.66 ± 37.54 | 3329.34 ± 35.62 | N/A | N/A | N/A | N/A |
| Wang 2022  (mean ± SEM | 3634.6 ± 37.5 | 3329 ± 35.6 | N/A | N/A | N/A | N/A |

Table 2.3 shows offspring data collected: Birth weight, Birth length and atopic profile where measured

**Appendix 7 - GDM Criteria and Management**

Most of the studies (7/8) adopted the International Association for Diabetes and Pregnancy Study Group Criteria (IASPSG) as the diagnostic criteria for determining GDM whereby patients are required to undertake a 2-hour 75g oral glucose tolerance test (OGTT). The full criteria is defined as: fasting glucose of ≥ 92 mg/dL or 1-hour-post 75 g-load ≥180 mg/dL or 2-hour glucose value ≥153 mg/dL.

(Yang et al., 2018) adopted the Carpenter and Coustan criteria which requires a 3-hour OGTT. The full criteria is defined as two or more values above the following are defined as GDM: fasting glucose ≥ 95, 1-hour ≥ 180, 2-hour ≥ 155, and 3-hour ≥ 140 mg/dL.

It is important to note that (Sheu et al., 2018) originally used the Australian Diabetes in Pregnancy criteria (ADIPS) defined as fasting plasma glucose ≥5.5 mmol/l (99 mg/dL) and/or 2 h plasma glucose ≥8.0 mmol/l (144 mg/dL) on a 2 h 75 g oral GTT. Women without co-morbidities were required to take 1-h 50g oral glucose challenge test (OGCT). All women with co-morbidities and those with a plasma glucose ≥7.8 mmol/l (140.4 mg/dL) on the GCT proceeded to a 2-h 75 g OGTT. Criteria for patients with co-morbidities/increased risk of GDM were defined as: a history of GDM in prior pregnancy, polycystic ovarian syndrome, BMI ≥35 kg/m2, maternal age ≥40 years or a first degree relative with type 2 diabetes were given the 2-h 75g OGTT regardless. In January 2015, they switched to using the IASPSG 2-h 75g OGTT for all patients and 44 of the 55 women with GDM fulfilled both criteria for the ADIPS and the IADPSG.

Management status of women with GDM was defined in three studies. The study (Sifnaios et al., 2019) excluded women with GDM who were using insulin and it was clearly stated that women with GDM were diet controlled and monitored throughout the study. (Schober et al., 2014) grouped women with GDM as diet-controlled (n = 21) and Insulin-dependent (n = 40) and sampled Tregs separately for these groups. (Zhao et al., 2020) documented the number of women with diet-controlled GDM (n = 16) and those managing with medications (n = 12) but did not differentiate between the groups when sampling Treg populations.

**Appendix 8 – Additional findings by the studies.**

In addition to measuring the proportion of Tregs, (Schober et al., 2014) measured Treg subsets and the suppressive activity of the Treg pool using CD4^+^CD127^LOW+/-^CD25^+^T_reg_; this was found to be moderately decreased in women with GDM.

Meanwhile, (Sheu et al., 2018) looked at T helper cell populations and compared ratios of T helper cells to Tregs. (Lobo et al., 2018) looked at Treg and natural killer cell profile as well as cytokine and chemokine production in Tregs. The study found a lower frequency of CD25^bright^ and FoxP3^high^ whereas higher TNF-A production by Tregs in GDM group was found.

Similarly, (Fagninou et al., 2020) also measured serum IL-10 levels as well as Th1 and Th2 ratios in addition to natural killer cells and monocytes. Serum Il-10 levels were lower in GDM while total CD4+ cell frequencies were higher in women with GDM.

(Yang et al., 2020) also measured serum cytokine levels in addition to Treg populations. Tregs were also measured as an absolute number of CD4+CD25+ T cells per ml of blood (also lower in women with GDM). GDM presented higher serum IL-6 and TNF-α levels and presented reduced levels of IL-10 and TGF-β.

Both (Zhao et al., 2020) and (Wang et al., 2022) also looked at CD8+ cells – no significant difference. Wang et al., also looked at CD8+ Tregs in addition to CD4+ Tregs. CD8+ Tregs were found to be higher in women with GDM. Wang et al., also included non-pregnant controls and pregnant women in their first trimester.

(Sifnaios et al., 2019) found that women with GDM had a higher proportion of Th2 and Th17 cells (as well as Treg cells) and exhibited no significant change in Th1/Th2/Th17/Treg profile post-partum (contrary to the control group).

This also highlights the heterogeneity in study aims and the immune profiles measured. All relevant data is available in table 3.
